# Prevalence of and Factors Associated With Nurse Burnout in the US

**DOI:** 10.1001/jamanetworkopen.2020.36469

**Published:** 2021-02-04

**Authors:** Megha K. Shah, Nikhila Gandrakota, Jeannie P. Cimiotti, Neena Ghose, Miranda Moore, Mohammed K. Ali

**Affiliations:** 1 Department of Family and Preventive Medicine, Emory University School of Medicine, Atlanta, Georgia; 2 Nell Hodgson Woodruff School of Nursing, Emory University, Atlanta, Georgia; 3 Department of Global Health, Rollins School of Public Health, Emory University, Atlanta, Georgia

## Abstract

**Importance:**

Clinician burnout is a major risk to the health of the US. Nurses make up most of the health care workforce, and estimating nursing burnout and associated factors is vital for addressing the causes of burnout.

**Objective:**

To measure rates of nurse burnout and examine factors associated with leaving or considering leaving employment owing to burnout.

**Design, Setting, and Participants:**

This secondary analysis used cross-sectional survey data collected from April 30 to October 12, 2018, in the National Sample Survey of Registered Nurses in the US. All nurses who responded were included (N = 3 957 661). Data were analyzed from June 5 to October 1, 2020.

**Exposures:**

Age, sex, race and ethnicity categorized by self-reported survey question, household income, and geographic region. Data were stratified by workplace setting, hours worked, and dominant function (direct patient care, other function, no dominant function) at work.

**Main Outcomes and Measures:**

The primary outcomes were the likelihood of leaving employment in the last year owing to burnout or considering leaving employment owing to burnout.

**Results:**

The 3 957 661 responding nurses were predominantly female (90.4%) and White (80.7%); the mean (weighted SD) age was 48.7 (0.04) years. Among nurses who reported leaving their job in 2017 (n = 418 769), 31.5% reported burnout as a reason, with lower proportions of nurses reporting burnout in the West (16.6%) and higher proportions in the Southeast (30.0%). Compared with working less than 20 h/wk, nurses who worked more than 40 h/wk had a higher likelihood identifying burnout as a reason they left their job (odds ratio, 3.28; 95% CI, 1.61-6.67). Respondents who reported leaving or considering leaving their job owing to burnout reported a stressful work environment (68.6% and 59.5%, respectively) and inadequate staffing (63.0% and 60.9%, respectively).

**Conclusions and Relevance:**

These findings suggest that burnout is a significant problem among US nurses who leave their job or consider leaving their job. Health systems should focus on implementing known strategies to alleviate burnout, including adequate nurse staffing and limiting the number of hours worked per shift.

## Key Points

**Question:**

What were the most recent US national estimates of nurse burnout and associated factors that may put nurses at risk for burnout?

**Findings:**

This secondary analysis of cross-sectional survey data from more than 3.9 million US registered nurses found that among nurses who reported leaving their current employment (9.5% of sample), 31.5% reported leaving because of burnout in 2018. The hospital setting and working more than 20 hours per week were associated with greater odds of burnout.

**Meaning:**

With increasing demands placed on frontline nurses during the coronavirus disease 2019 pandemic, these findings suggest an urgent need for solutions to address burnout among nurses.

## Teaser

This secondary analysis of a cross-sectional survey measures burnout rates and examines factors associated with leaving or considering leaving employment owing to burnout among US nurses.

## Introduction

Clinician burnout is a threat to US health and health care.^1^ At more than 6 million in 2019,^2^ nurses are the largest segment of our health care workforce, making up nearly 30% of hospital employment nationwide.^3^ Nurses are a critical group of clinicians with diverse skills, such as health promotion, disease prevention, and direct treatment. As the workloads on health care systems and clinicians have grown, so have the demands placed on nurses, negatively affecting the nursing work environment. When combined with the ever-growing stress associated with the coronavirus disease 2019 (COVID-19) pandemic, this situation could leave the US with an unstable nurse workforce for years to come. Given their far-ranging skill set, importance in the care team, and proportion of the health care workforce, it is imperative that we better understand job-related outcomes and the factors that contribute to burnout in nurses nationwide.

Demanding workloads and aspects of the work environment, such as poor staffing ratios, lack of communication between physicians and nurses, and lack of organizational leadership within working environments for nurses, are known to be associated with burnout in nurses.^4,5^ However, few, if any, recent national estimates of nurse burnout and contributing factors exist. We used the most recent nationally representative nurse survey data to characterize burnout in the nurse workforce before COVID-19. Specifically, we examined to what extent aspects of the work environment resulted in nurses leaving the workforce and the factors associated with nurses’ intention to leave their jobs and the nursing profession.

## Methods

### Data Source

We used data from the 2018 US Department of Health and Human Services’ Health Resources and Service Administration National Sample Survey of Registered Nurses (NSSRN), a nationally representative anonymous sample of registered nurses in the US. The weighted response rate for the 2018 NNRSN is estimated at 49.0%.^6^ Details on sampling frame, selection, and noninterview adjustments are described elsewhere.^7^ Weighted estimates generalize to state and national nursing populations.^6^ The American Association for Public Opinion Research Response Rate 3 method was used to calculate the NSSRN response rate.^6^ This study of deidentified publicly available data was determined to be exempt from approval and informed consent by the institutional review board of Emory University. This study followed the Strengthening the Reporting of Observational Studies in Epidemiology (STROBE) reporting guideline for cross-sectional studies

### Variables and Definitions

Data were collected from April 30 to October 12, 2018. We generated demographic characteristics from questions about years worked in the profession, primary and secondary nursing positions, and work environment. We included the work environment variables of primary employment setting and full-time or part-time status. We grouped responses to a question on dominant nursing tasks as direct patient care, other, and no dominant task. We included 3 categories of educational attainment (diploma/ADN, BSN, or MSN/PhD/DNP degrees) and whether the respondent was internationally educated. Other variables included change in employment setting in the last year, hours worked per week, and reasons for employment change.

We categorized employment setting as (1) hospital (not mental health), (2) other inpatient setting, (3) clinic or ambulatory care, and (4) other types of setting. Workforce stability was defined as the percentage of nurses with less than 5 years of experience in the nursing profession.

We used 2 questions to assess burnout and other reasons for leaving or planning to leave a nursing position. Nurses who had left the position they held on December 31, 2017, were asked to identify the reasons contributing to their decision to leave their prior position. Nurses who were still employed in the position they held on December 31, 2017, and answered yes to the question “Have you ever considered leaving the primary nursing position you held on December 31, 2017?” were asked “Which of the following reasons would contribute to your decision to leave your primary nursing position?”

### Statistical Analysis

Data were analyzed from June 5 to October 1, 2020. We used descriptive statistics to characterize nurse survey responses. For continuous variables, we reported means and SDs and for categorical variables, frequencies (number [percentage]). Further, we examined the overlap of the proportions who reported leaving or considered leaving their job owing to burnout and other factors. We then fit 2 separate logistic regression models to estimate the odds that aspects of the work environment, hours, and tasks were associated with the following outcomes related to burnout: (1) left job owing to burnout and (2) considered leaving their job owing to burnout. We controlled for nurse demographic characteristics of age, sex, race, household income, and geographic region and reported odds ratios (ORs) and 95% CIs. Two separate sensitivity analyses were performed: (1) we used a broader theme of burnout defined as a response of burnout, inadequate staffing, or stressful work environment for the regression models; and (2) we stratified the regression models by respondents younger than 45 years and 45 years or older to examine difference by age.

We used SAS, version 9.4 (SAS Institute, Inc), with statistical significance set at 2-sided α = .05. We used sample weights to account for the differential selection probabilities and nonresponse bias.

## Results

The 3 957 661 nurse respondents in 2018 were mostly female (90.4%) and White (80.7%). The mean (weighted SD) age of nurse respondents was 48.7 (0.04) years, and 95.3% were US graduates. The percentage of nurses with a BSN degree was 45.8%; with an MSN, PhD, or DNP degree, 16.3%; and 49.5% of nurses reported that they worked in a hospital. The mean (weighted SD) age of nurses who left their job due to burnout was 42.0 (0.6) years; for those considering leaving their job due to burnout, 43.7 (0.3) years (Table 1).

**Table 1.** Demographic Characteristics of Respondents of the 2018 National Sample Survey of Registered Nurses

Variable	Respondent sample^a^		
	All (N = 3 957 661)	Nurses who left their job owing to burnout (n = 131 757)	Nurses considering leaving their job owing to burnout (n = 676 122)
Age, mean (weighted SD), y	48.7 (0.04)	42.0 (0.6)	43.7 (0.3)
Sex			
Female	3 577 407 ± 2437 (90.4)	119 654 ± 6570 (90.81)	603 103 ± 13 928 (89.2)
Male	380 254 ± 2437 (9.6)	12 103 ± 2157 (9.2)	73 019 ± 5397 (10.8)
Marital status			
Now married	2 801 108 ± 17 169 (70.8)	84 419 ± 5604 (64.1)	459 595 ± 12 223 (68.0)
Widowed, divorced, separated	646 076 ± 12 459 (16.3)	24 867 ± 3881 (18.9)	91 461 ± 5979 (13.5)
Never married	510 477 ± 11 813 (12.9)	22 471 ± 3325 (17.1)	125 067 ± 6542 (18.5)
Race/ethnicity			
White only	3 192 598 ± 9843 (80.7)	104 710 ± 6423 (79.4)	550 587 ± 13 181 (81.4)
Black only	318 884 ± 3613 (8.1)	11 600 ± 2324 (8.8)	53 631 ± 4593 (7.9)
Asian/Pacific Islander	237 258 ± 5193 (6.0)	6524 ± 1660 (5.0)	604 218 ± 4679 (5.3)
American Indian/Alaska Native only	17 863 ± 2913 (0.5)	583.74 ± 356.64 (0.4)	3255 ± 1268 (0.5)
Other	191 058 ± 9583 (4.8)	8339 ± 2104 (6.3)	32 849 ± 3260 (6.3)
Income			
≤$50 000	377 868 ± 15 071 (9.6)	13 661.22 ± 2297 (10.4)	36 620 ± 3226 (5.4)
$50 001-$75 000	727 424 ± 13 301 (18.4)	33 013 ± 4139 (25.1)	135 952 ± 7695 (20.1)
$75 001-$100 000	874 244 ± 15 184 (22.1)	31 803 ± 3299 (24.1)	152 546 ± 6537 (22.6)
$100 001-$150 000	1 109 983 ± 13 899 (28.1)	33 585 ± 3143 (25.5)	220 982 ± 9208 (32.7)
>$150 000	868 142 ± 13 607 (21.9)	19 696 ± 2402 (15.0)	130 022 ± 6735 (19.2)
Graduate program			
US	3 769 870 ± 8001 (95.3)	128 440 ± 7148 (97.5)	657 128 ± 15 295 (97.2)
Outside US	187 791 ± 8001 (4.7)	3318 ± 1113 (2.5)	18 995 ± 2707 (2.8)
Work setting			
Clinic/ambulatory^b^	511 847 ± 10 619 (12.9)	11 291 ± 1662 (8.6)	76 271 ± 4240 (11.3)
Hospital^c^	1 959 308 ± 16 988 (49.5)	83 971 ± 5856 (63.7)	466 750 ± 13 787 (69.0)
Other inpatient setting^d^	270 493 ± 9331 (6.8)	18 800 ± 2489 (14.3)	51 655 ± 4297 (7.6)
Other types of setting^e^	531 223 ± 11 352 (13.4)	17 695 ± 2507 (13.4)	81 446 ± 5815 (12.1)
Employment setting compared with previous year			
Same	3 057 942 ± 14 221 (77.3)	112 223 ± 6710 (89.8)	629 945 ± 14 956 (96.5)
Different	188 721 ± 6951 (4.8)	12 813 ± 2238 (10.3)	22 708 ± 1964 (3.5)
Highest nursing education			
Diploma/ADN	1 499 372 ± 17 639 (37.9)	53 354 ± 4866 (40.5)	228 956 ± 8455 (33.9)
BSN	1 809 156 ± 18 949 (45.8)	62 562 ± 4811 (47.5)	341 194 ± 11 902 (50.5)
MSN/PhD/DNP	644 785 ± 11 377 (16.3)	15 841 ± 1476 (12.0)	105 582 ± 4731 (15.6)
Employment status			
Employed in nursing full-time	2 583 193 ± 20 524 (65.3)	108 058 ± 6715 (82.0)	564 000 ± 15 398 (83.4)
Employed in nursing part-time	689 679 ± 14 733 (17.4)	23 699 ± 3707 (18.0)	112 122 ± 5228 (16.6)
Not employed in nursing	684 789 ± 12 514 (17.3)	-	-
Dominant function at workplace			
Direct patient care	1 949 826 ± 17 829 (59.6)	78 415 ± 5524 (59.5)	417 918 ± 10 582 (61.8)
Other function^f^	583 001 ± 11 988 (17.8)	16 730 ± 2278 (12.7)	95 236 ± 6389 (14.1)
No dominant function	740 044 ± 15 156 (22.6)	36 613 ± 3792 (27.8)	162 967 ± 8714 (24.1)
Hours worked per week, mean (SD)	37.5 (0.1)	40.3 (0.7)	38.8 (0.2)
Region of practice			
Northeast	624 060 ± 6323 (19.1)	21 748 ± 2481 (16.5)	134 150 ± 6250 (19.8)
South	1 185 863 ± 9242 (36.2)	53 119 ± 4948 (40.3)	235 190 ± 9063 (34.8)
Midwest	806 048 ± 7983 (24.6)	32 714 ± 3196 (24.8)	174 180 ± 6158 (25.8)
West	656 900 ± 8568 (20.1)	24 175 ± 3065 (18.4)	132 602 ± 7654 (19.6)

^a^ Unless otherwise indicated, data are expressed as number ± CIs for weighting (weighted percentage) of respondents.

^b^ Includes private medical practice (clinic, physician office, etc), public clinic (rural health center, federally qualified health center, Indian Health Service, tribal clinic, etc), school health service (kindergarten through 12th grade or college), nurse-managed health center, outpatient mental health/substance abuse facility, urgent care clinic, and ambulatory surgery center.

^c^ Includes hospital-sponsored ambulatory care (outpatient, surgery, clinic, urgent care, etc), hospital ancillary unit, hospital nursing home unit, Critical Access Hospital (a rural community hospital that receives cost-based reimbursement from Medicare), inpatient unit, emergency department, and hospital administration.

^d^ Includes correctional facility, inpatient hospice, nursing home unit not in hospital, rehabilitation facility/long-term care, and inpatient mental health/substance abuse facility.

^e^ Includes home health agency/service, occupational health or employee health service, public health agency (not a clinic), government agency other than public/community health or correctional facility, outpatient dialysis center, university or college academic department, insurance company, and call center/telenursing center.

^f^ Includes administrative work, research, teaching, supervision, and nonnursing tasks.

**Figure 1.**
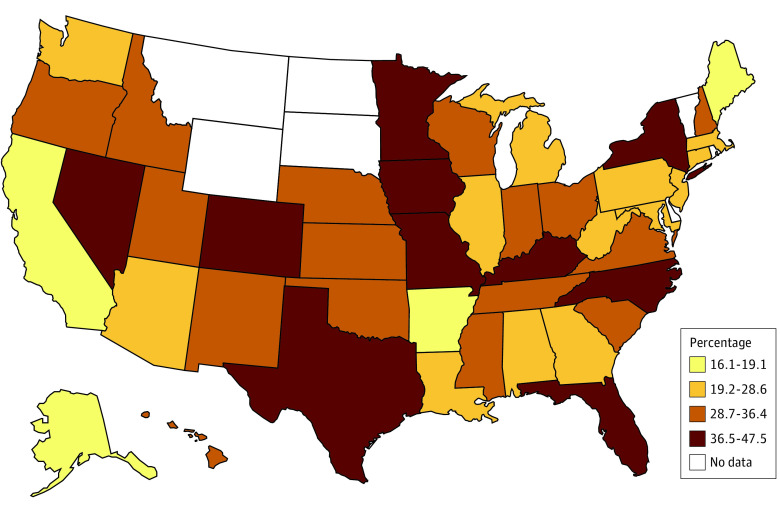
State-Level Distribution of Nurses Who Left Their Jobs Owing to Burnout Data are from the 2018 National Sample Survey of Registered Nurses.

**Figure 2.**
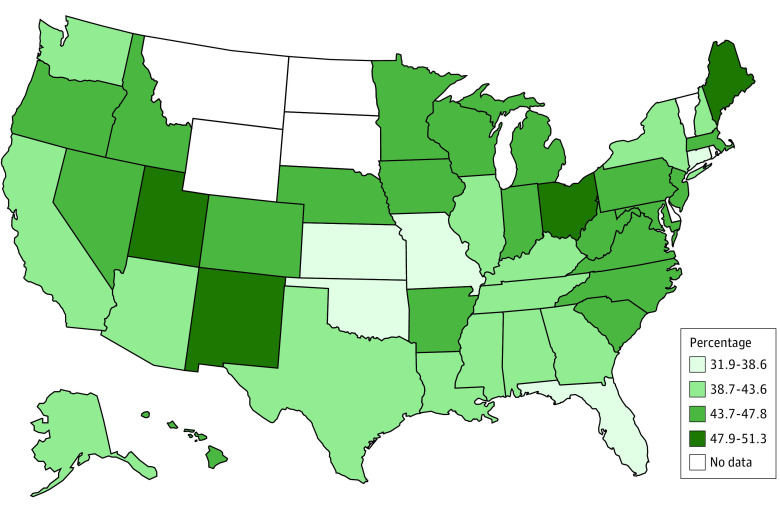
State-Level Distribution of Nurses Who Considered Leaving Their Jobs Owing to Burnout Data are from the 2018 National Sample Survey of Registered Nurses.

**Figure 3.**
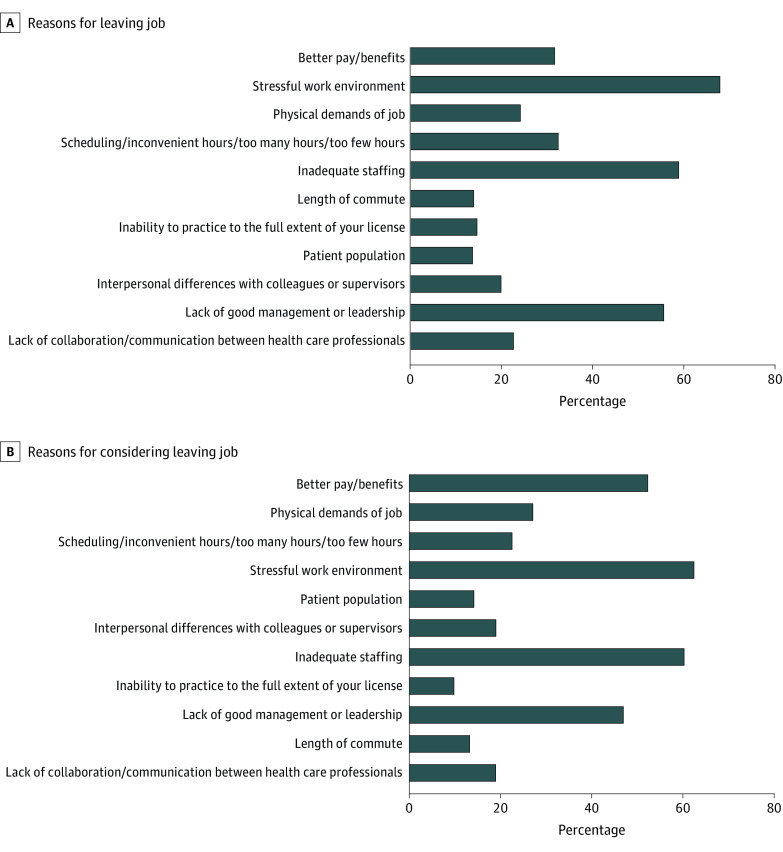
Overlap of Work Reasons for Nurses Who Left or Considered Leaving Their Jobs Owing to Burnout Data are from the 2018 National Sample Survey of Registered Nurses.

Of the total sample of nurses (N = 3 957 661), 9.5% reported leaving their most recent position (n = 418 769), and of those, 31.5% reported burnout as a reason contributing to their decision to leave their job (3.3% of the total sample) (eTable in the Supplement). For nurses who had considered leaving their position (n = 676 122), 43.4% identified burnout as a reason that would contribute to their decision to leave their current job. Additional factors in these decisions were a stressful work environment (34.4% as the reason for leaving and 41.6% as the reason for considering leaving), inadequate staffing (30.0% as the reason for leaving and 42.6% as the reason for considering leaving), lack of good management or leadership (33.9% as the reason for leaving and 39.6% as the reason for considering leaving), and better pay and/or benefits (26.5% as the reason for leaving and 50.4% as the reason for considering leaving). By geographic regions of the US, lower proportions of nurses reported burnout in the West (16.6%), and higher proportions reported burnout in the Southeast (30.0%) (Figure 1 and Figure 2). Figure 3 shows the overlap between leaving or considering leaving their position owing to burnout and other reasons. For both outcomes, the highest overlap response with burnout was for stressful work environment (68.6% of those who left their job and 63.0% of those who considered leaving their job due to burnout).

The adjusted regression models estimating the odds of nurses indicating burnout as a reason for leaving their positions or considering leaving their position revealed statistically significant associations between workplace settings and hours worked per week, but not for tasks performed, and burnout (Table 2). For nurses who had left their jobs, compared with nurses working in a clinic setting, nurses working in a hospital setting had more than twice higher odds of identifying burnout as a reason for leaving their position (OR, 2.10; 95% CI, 1.41-3.13); nurses working in other inpatient settings had an OR of 2.26 (95% CI, 1.39-3.68). Compared with working less than 20 h/wk, nurses who worked more than 40 h/wk had an OR of 3.28 (95% CI, 1.61-6.67) for identifying burnout as a reason they left their position.

**Table 2.** Multivariate Regression Analyses Estimating the Odds of Leaving and Planning to Leave Job Owing to Burnout^a^

Variable	OR (95% CI)
**Left job owing to burnout (n = 131 757)**	
Workplace setting	
Clinic^b^	1 [Reference]
Hospital^c^	2.10 (1.41-3.13)
Other inpatient^d^	2.26 (1.39-3.68)
Other settings^e^	1.33 (0.82-2.16)
Hours worked per week	
<20	1 [Reference]
21-30	2.11 (0.95-4.71)
31-40	2.26 (1.19-4.29)
>40	3.28 (1.61-6.67)
Dominant function	
Direct patient care	1 [Reference]
Other^f^	0.67 (0.47-0.95)
None	1.29 (0.96-1.72)
**Considered leaving job owing to burnout (n = 676 122)**	
Workplace setting	
Clinic^b^	1 [Reference]
Hospital^c^	1.80 (1.55-2.08)
Other inpatient^d^	1.35 (1.05-1.73)
Other settings^e^	0.98 (0.77-1.25)
Hours worked per week	
<20	1 [Reference]
21-30	2.56 (1.85-3.55)
31-40	2.98 (2.24-3.98)
>40	3.64 (2.73-4.85)
Dominant function	
Direct patient care	1 [Reference]
Other^f^	0.75 (0.63-0.90)
None	1.03 (0.88-1.20)

Abbreviation: OR, odds ratio.

^a^ Data are from the 2018 National Sample Survey of Registered Nurses. Analyses are adjusted for age, sex, race/ethnicity, household income, and region.

^b^ Includes private medical practice (clinic, physician office, etc), public clinic (rural health center, federally qualified health center, Indian Health Service, tribal clinic, etc), school health service (kindergarten through 12th grade or college), nurse-managed health center, outpatient mental health/substance abuse facility, urgent care clinic, and ambulatory surgery center.

^c^ Includes hospital-sponsored ambulatory care (outpatient, surgery, clinic, urgent care, etc), hospital ancillary unit, hospital nursing home unit, Critical Access Hospital (a rural community hospital that receives cost-based reimbursement from Medicare), inpatient unit, emergency department, and hospital administration.

^d^ Includes correctional facility, inpatient hospice, nursing home unit not in hospital, rehabilitation facility/long-term care, and inpatient mental health/substance abuse facility.

^e^ Includes home health agency/service, occupational health or employee health service, public health agency (not a clinic), government agency other than public/community health or correctional facility, outpatient dialysis center, university or college academic department, insurance company, and call center/telenursing center.

^f^ Includes administrative work, research, teaching, supervision, and nonnursing tasks.

For nurses who reported ever considering leaving their job, working in a hospital setting was associated with 80% higher odds of burnout as the reason than for nurses working in a clinic setting (OR, 1.80; 95% CI, 1.55-2.08), whereas among nurses who worked in other inpatient settings, burnout was associated with a 35% higher odds that nurses intended to leave their job (OR, 1.35; 95% CI, 1.05-1.73). Compared with working less than 20 h/wk, the odds of identifying burnout as a reason for considering leaving their position increased with working 20 to 30 h/wk (OR, 2.56; 95% CI, 1.85-3.55), 31 to 40 h/wk, (OR, 2.98; 95% CI, 2.24-3.98), and more than 40 h/wk, (OR, 3.64; 95% CI, 2.73-4.85).

The sensitivity analysis results in which a broader classification of burnout was used showed a similar relationship between odds of burnout and working more than 40 h/wk (OR, 3.86; 95% CI, 2.27-6.59) for those who left their job (OR, 2.66; 95% CI, 2.13-3.31). Stratification by those younger than 45 years and 45 years or older did not significantly change the findings. Figure 3 shows the overlap in nurses who reported burnout and other reasons for leaving their current position or considering leaving their current positions. The greatest overlap occurred in responses of burnout and stressful work environment (68.6% of those who reported leaving and 59.5% of those who considered leaving) and inadequate staffing (63.0% of those who reported leaving and 60.9% of those who considered leaving).

## Discussion

Our findings from the 2018 NSSRN show that among those nurses who reported leaving their jobs in 2017, high proportions of US nurses reported leaving owing to burnout. Hospital setting was associated with greater odds of identifying burnout in decisions to leave or to consider leaving a nursing position, and there was no difference by dominant work function.

Health care professionals are generally considered to be in one of the highest-risk groups for experience of burnout, given the emotional strain and stressful work environment of providing care to sick or dying patients.^8,9^ Previous studies demonstrate that 35% to 54% of clinicians in the US experience burnout symptoms.^10,11,12,13^ The recent National Academy of Medicine report, “Taking Action Against Clinician Burnout: A Systems Approach to Professional Well-Being,” recommended health care organizations routinely measure and monitor clinician burnout and hold leaders accountable for the health of their organization’s work environment in an effort to reduce burnout and promote well-being.^1^

Moreover, it appears the numbers have increased over time. Data from the 2008 NSSRN showed that approximately 17% of nurses who left their position in 2007 cited burnout as the reason for leaving,^14^ and our data show that 31.5% of nurses cited burnout as the reason for leaving their job in the last year (2017-2018). Despite this evidence, little has changed in health care delivery and the role of registered nurses. The COVID-19 pandemic has further complicated matters; for example, understaffing of nurses in New York and Illinois was associated with increased odds of burnout amidst high patient volumes and pandemic-related anxiety.^15^

Our findings show that among nurses who reported leaving their job owning to burnout, a high proportion reported a stressful work environment. Substantial evidence documents that aspects of the work environment are associated with nurse burnout. Increased workloads, lack of support from leadership, and lack of collaboration among nurses and physicians have been cited as factors that contribute to nurse burnout.^4,16^ Magnet hospitals and other hospitals with a reputation for high-quality nursing care have shown that transforming features of the work environment, including support for education, positive physician-nurse relationships, nurse autonomy, and nurse manager support, outside of increasing the number of nurses, can lead to improvements in job satisfaction and lower burnout among nurses.^17,18,19^ The qualities of Magnet hospitals not only attract and retain nurses and result in better nurse outcomes, based on features of the work environment, but also improvements in the overall quality of patient care.^17,18,19^

Self-reported regional variation in burnout deserves attention. The lower reported rates of nurse burnout in California and Massachusetts could be attributed to legislation in these states regulating nurse staffing ratios; California has the most extensive nurse staffing legislation in the US.^20^ The high rates of reported burnout in the Southeast and the overlap of burnout and inadequate staffing in our findings could be driven by shortages of nurses in the states in this area, particularly South Carolina and Georgia.^15^ Geographic distribution, nurse staffing, and its association with self-reported burnout warrant further exploration.

Our data show that the number of hours worked per week by nurses, but not the dominant function at work, was positively associated with identifying burnout as a reason for leaving their position or considering leaving their position. Research suggests nurses who work longer shifts and who experience sleep deprivation are likely to develop burnout.^21,22,23^ Others have reported a strong correlation between sleep deprivation and errors in the delivery of patient care.^22,24^ Emotional exhaustion has been identified as a major component of burnout; such exhaustion is likely exacerbated by excessive work hours and inadequate sleep.^25,26^

The nurse workforce represents most current frontline workers providing care during the COVID-19 pandemic. Literature from past epidemics (eg, H1N1 influenza, severe acute respiratory syndrome, Ebola) suggest that nurses experience significant stress, anxiety, and physical effects related to their work.^27^ These factors will most certainly be amplified during the current pandemic, placing the nurse workforce at risk of increased strain. Recent reports suggest that nurses are leaving the bedside owing to COVID-19 at a time when multiple states are reporting a severe nursing shortage.^28,29,30,31^ Furthermore, given that the nurse workforce is predominantly female and married, the child rearing and domestic responsibilities of current lockdowns and quarantines can only increase their burden and risk of burnout. Our results demonstrate that the mean age at which nurses who have left or considered leaving their current jobs is younger than 45 years. In the present context, our results forewarn of major effects to the frontline nurse workforce. Further studies are needed to elucidate the effect of the current pandemic on the nurse workforce, particularly among younger nurses of color, who are underrepresented in these data. Policy makers and health systems should also focus on aspects of the work environment known to improve job satisfaction, including staffing ratios, continued nursing education, and support for interdisciplinary teamwork.

### Limitations

Our study has some limitations. First, our findings are from cross-sectional data and limit causal inference; however, these data represent the most recent and, to our knowledge, the only national survey with data on nurse burnout. Second, our burnout measure is crude, and more extensive measures of burnout are needed. Third, 4 states did not have enough respondents to release data (Montana, Wyoming, North Dakota, and South Dakota). However, these data were weighted, and they represent the most comprehensive data available on the registered nurse workforce. Fourth, nonresponse analyses of these data reveal underestimation of certain races/ethnicities, specifically Hispanic nurses, and small sample sizes limited analyses of burnout by race/ethnicity. Fifth, the public use file of the NSSRN does not disaggregate the MSN, PhD, and DNP degrees in nursing practice categories. Given that these job tasks can vary, we addressed this limitation by examining dominant function at work. Last, the response rate was modest at 49.0% (weighted). Despite these limitations, this analysis is most likely the first to provide an updated overview of registered nurse burnout across the US.

## Conclusions

Burnout continues to be reported by registered nurses across a variety of practice settings nationwide. How the COVID-19 pandemic will affect burnout rates owing to unprecedented demands on the workforce is yet to be determined. Legislation that supports adequate staffing ratios is a key part of a multitiered solution. Solutions must come through system-level efforts in which we reimagine and innovate workflow, human resources, and workplace wellness to reduce or eliminate burnout among frontline nurses and work toward healthier clinicians, better health, better care, and lower costs.^32^

## Notes

Supplement. **eTable.** Top 5 Reasons for Leaving Job and Considering Leaving Job by Respondents, 2018 National Sample Survey of Registered Nurses
