# Error in Sample Sizes

**DOI:** 10.1001/jamanetworkopen.2021.5373

**Published:** 2021-03-16

In the Original Investigation titled “Prevalence of and Factors Associated With Nurse Burnout in the US,”^1^ published February 4, 2021, the Key Points, Abstract, Results, and Table 1 mistakenly identified the number of survey respondents as 3 957 661; in fact, this was a weighted value based on a smaller number of survey responses. This article has been corrected.^1^
