# Error in Funding/Support

**DOI:** 10.1001/jamanetworkopen.2023.12593

**Published:** 2023-04-25

In the Original Investigation titled “Prevalence of and Factors Associated With Nurse Burnout in the US,”^1^ published February 4, 2021, the Funding/Support section was updated to add a previously missing grant awarded to Dr Cimiotti. This article has been corrected.^1^
