# Supplementary material for: Prevalence of and Factors Associated With Nurse Burnout in the US
Source: JAMA Netw Open. 2021 Feb 4;4(2):e2036469. doi: 10.1001/jamanetworkopen.2020.36469 (PMC7862989; doi:10.1001/jamanetworkopen.2020.36469)
Supplement: Supplement. — eTable. Top 5 Reasons for Leaving Job and Considering Leaving Job by Respondents, 2018 National Sample Survey of Registered Nurses [file jamanetwopen-e2036469-s001.pdf]

Supplementary Online Content

Shah MK, Gandrakota N, Cimiotti JP, Ghose N, Moore M, Ali MK. Prevalence of and factors associated with nurse burnout in the US. *JAMA Netw Open*. 2021;4(2): e2036469. doi:10.1001/jamanetworkopen.2020.36469

eTable. Top 5 Reasons for Leaving Job and Considering Leaving Job by Respondents, 2018 National Sample Survey of Registered Nurses

This supplementary material has been provided by the authors to give readers additional information about their work.

©2021 Shah MK et al. *JAMA Network Open*.

eTable. Top 5 Reasons for Leaving Job and Considering Leaving Job by Respondents, 2018 National Sample Survey of Registered Nurses

**Reasons for leaving job (n = 418,769)**		**No.**	**Percentage**
1	Stressful work environment	144,017	34%
2	Lack of good management or leadership	141,764	34%
3	Burnout	131,757	31%
4	Inadequate Staffing	125,836	30%
5	Better Pay/Benefits	110,943	26%
**Reasons for considering to leave job (n = 1,558,631)**			
1	Better Pay/Benefits	785,679	50%
2	Burnout	676,122	43%
3	Inadequate Staffing	663,469	43%
4	Stressful work environment	648,155	42%
5	Lack of good management or leadership	617,368	40%

*Categories are not mutually exclusive

©2021 Shah MK et al. *JAMA Network Open*.
